# DRP-1 is required for BH3 mimetic-mediated mitochondrial fragmentation and apoptosis

**DOI:** 10.1038/cddis.2016.485

**Published:** 2017-01-12

**Authors:** Mateus Milani, Dominic P Byrne, Georgia Greaves, Michael Butterworth, Gerald M Cohen, Patrick A Eyers, Shankar Varadarajan

**Affiliations:** 1 Department of Molecular and Clinical Cancer Medicine, Institute of Translational Medicine, University of Liverpool, Ashton Street, Liverpool L69 3GE, UK; 2 Department of Biochemistry, Institute of Integrative Biology, University of Liverpool, Crown Street, Liverpool L69 7ZB, UK; 3 Department of Molecular and Clinical Pharmacology, Institute of Translational Medicine, University of Liverpool, Liverpool, Ashton Street, Liverpool L69 3GE, UK

## Abstract

The concept of using BH3 mimetics as anticancer agents has been substantiated by the efficacy of selective drugs, such as Navitoclax and Venetoclax, in treating BCL-2-dependent haematological malignancies. However, most solid tumours depend on MCL-1 for survival, which is highly amplified in multiple cancers and a major factor determining chemoresistance. Most MCL-1 inhibitors that have been generated so far, while demonstrating early promise *in vitro*, fail to exhibit specificity and potency in a cellular context. To address the lack of standardised assays for benchmarking the *in vitro* binding of putative inhibitors before analysis of their cellular effects, we developed a rapid differential scanning fluorimetry (DSF)-based assay, and used it to screen a panel of BH3 mimetics. We next contrasted their binding signatures with their ability to induce apoptosis in a MCL-1 dependent cell line. Of all the MCL-1 inhibitors tested, only A-1210477 induced rapid, concentration-dependent apoptosis, which strongly correlated with a thermal protective effect on MCL-1 in the DSF assay. In cells that depend on both MCL-1 and BCL-X_L_, A-1210477 exhibited marked synergy with A-1331852, a BCL-X_L_ specific inhibitor, to induce cell death. Despite this selectivity and potency, A-1210477 induced profound structural changes in the mitochondrial network in several cell lines that were not phenocopied following MCL-1 RNA interference or transcriptional repression, suggesting that A-1210477 induces mitochondrial fragmentation in an MCL-1-independent manner. However, A-1210477-induced mitochondrial fragmentation was dependent upon DRP-1, and silencing expression levels of DRP-1 diminished not just mitochondrial fragmentation but also BH3 mimetic-mediated apoptosis. These findings provide new insights into MCL-1 ligands, and the interplay between DRP-1 and the anti-apoptotic BCL-2 family members in the regulation of apoptosis.

Targeting the diverse anti-apoptotic BCL-2 family of proteins offers substantial promise for cancer treatment and has the potential to be valuable in overcoming tumour recurrence and chemoresistance. In particular, the BCL-2 selective inhibitor, ABT-199 (Venetoclax) and ABT-263 (Navitoclax), which also targets BCL-2, BCL-X_L_ and BCL-w, have been employed successfully for treating haematological malignancies.^1, 2, 3^ However, these inhibitors are ineffective in treating solid tumours, whose survival often depends on the overexpression of the anti-apoptotic protein MCL-1. MCL-1 is one of the most widely expressed pathologic factors in human cancers,^4^ and many putative MCL-1 inhibitors have been synthesised, several of which have demonstrated selectivity in different types of *in vitro* assays.^5, 6, 7, 8, 9, 10, 11, 12, 13^

Inhibitors of the BCL-2 family of proteins, widely referred to as BH3 mimetics, elicit their pro-apoptotic roles by activating BAX and or BAK, which perturbs mitochondrial integrity resulting in the release of cytochrome *c* and caspase activation.^14^ The putative inhibitors of MCL-1 evaluated in the current study have all been designed to function as BH3 mimetics, and a variety of analytical data from different *in vitro* studies has demonstrated their ability to target MCL-1.^5, 6, 7, 8, 9, 10, 11, 12, 13^ However, the lack of a single benchmarked binding assay to evaluate compound binding and reproducibility has hindered compound comparisons, with most assays relying upon fluorescence polarisation, which is subject to signal-to-noise artefacts and potential interference from the compounds. Indeed, many described MCL-1 inhibitors have failed to enter clinical trials, potentially due to a lack of specificity and potency.

In this study, we purified recombinant human MCL-1 from bacteria and developed a rapid, simple differential scanning fluorimetry (DSF) assay, which we exploit to screen a broad panel of BH3 mimetics. Using a thermostability protocol, we validate A-1210477 as a potent and selective MCL-1 ligand *in vitro*. We corroborate our DSF assay in a series of cellular experiments, showing that A-1210477 induces rapid apoptosis in an MCL-1-dependent cell line. Furthermore, we observe rapid and extensive mitochondrial fission following A-1210477 that occurs in a DRP-1-dependent manner. Finally, we characterise roles for DRP-1 in A-1210477-mediated mitochondrial fragmentation as well as in BH3 mimetic-mediated apoptosis. Thus, our report identifies A-1210477 as a bona fide MCL-1 inhibitor that not only facilitates apoptosis but also grossly perturbs mitochondrial membrane dynamics.

## Results

### Most described MCL-1 inhibitors fail to potently induce apoptosis in a MCL-1 dependent cell line

**Figure 1:**
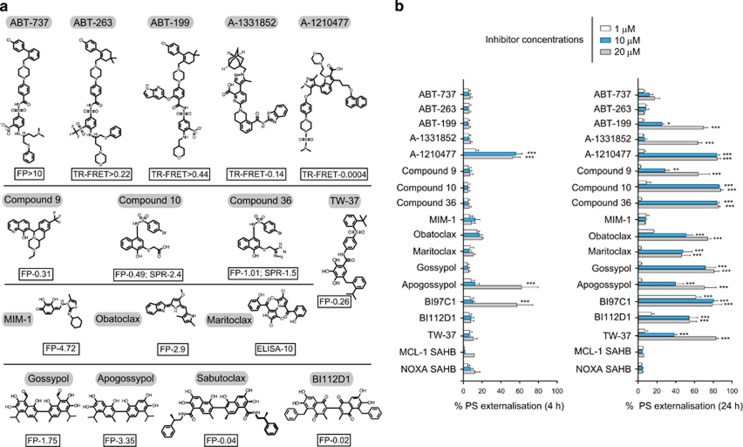
Reported *in vitro* binding constants of MCL-1 inhibitors correlate poorly with the ability to induce apoptosis in a cellular context. (**a**) Chemical structures of reported BH3 mimetics used in this study along with literature binding affinities (*μ*M), assessed by fluorescence polarisation (FP), surface plasmon resonance (SPR) or time-resolved fluorescence resonance energy transfer (TR-FRET). (**b**) Analysis of concentration-dependent effects of MCL-1 inhibitors on cellular apoptosis, assessed by the extent of phosphatidylserine (PS) externalisation following 4 and 24 h of exposure in MCL-1 addicted H929 cells. Statistical analysis was conducted using one-way ANOVA and *P*-values depicted as ***P⩽0.001. Results are shown as the mean±S.E.M. (standard error of the mean) from at least three independent experiments

The literature contains numerous examples of small molecule MCL-1 inhibitors that exhibit a wide range of reported binding affinities for MCL-1 (Figure 1). These have been calculated using a variety of *in vitro* approaches, including fluorescence polarisation (FP), surface plasmon resonance (SPR), ELISA and time-resolved fluorescence resonance energy transfer (TR-FRET; Figure 1). The first selective inhibitors of the BCL-2 family of proteins, ABT-737 and its orally available analogue, ABT-263 (Navitoclax) target BCL-2, BCL-X_L_ and BCL-w, but not MCL-1, at low nanomolar concentrations.^1^ These compounds have been followed by ABT-199 (Venetoclax), A-1331852 and A-1210477, which, respectively, target BCL-2, BCL-X_L_ and MCL-1.^2, 5, 15^ The MCL-1 ligand ‘Compound 9' was generated as a result of a HTS strategy coupled to direct hit optimisation,^6^ while MIM-1 was identified by a stapled peptide-based competitive screen.^7^ A series of 3-substituted-N-(4-Hydroxynaphthalen-1-yl) arylsulphonamides, including compounds 10 and 36, have been reported to bind and inhibit MCL-1.^8^ Obatoclax mesylate is a pan-BCL-2 inhibitor with reported specificity for MCL-1.^9^ Maritoclax (marinopyrrole A1) is a natural product that directly binds MCL-1 and targets it for proteasomal degradation.^10^ Removal of the toxic aldehyde groups in the naturally occurring polyphenol, gossypol, resulted in apogossypol, which upon further substitution yielded BI97C1 (Sabutoclax) and BI112D1, both of which are claimed to target all members of the BCL-2 family.^11, 12^ TW-37 is a benzenesulphonyl derivative of gossypol reported to bind to MCL-1 with a higher affinity than BCL-2 or BCL-X_L_ (Figure 1a).^13^

To investigate whether these compounds induce cellular apoptosis by inhibiting MCL-1, we exposed MCL-1-addicted H929 cells to this broad panel of putative inhibitors (Figure 1a) and assessed the extent of apoptosis induction. Also included in our panel as negative controls were ABT-737, ABT-263, ABT-199 and A-1331852, which were assessed alongside the stabilised alpha-helix of BCL-2 domains (SAHBs), which were designed to mimic the BH3 binding helix of MCL-1 and NOXA, respectively.^16^ Remarkably, only A-1210477 induced extensive concentration-dependent apoptosis in H929 cells following a brief (4 h) exposure (Figure 1b). Both apogossypol and BI97C1 (Sabutoclax) induced apoptosis at high micromolar concentrations, whereas all other inhibitors failed to exhibit any enhanced apoptosis (Figure 1b). In contrast, a prolonged exposure (24 h) to several compounds, including ABT-199, A-1331852, Obatoclax, Maritoclax, gossypol derivatives and compounds 9, 10 and 36, resulted in a marked increase in cell death (Figure 1b), suggesting that they function through an indirect or off-target effect.

### Design and validation of a new assay to evaluate MCL-1 compound binding

**Figure 2:**
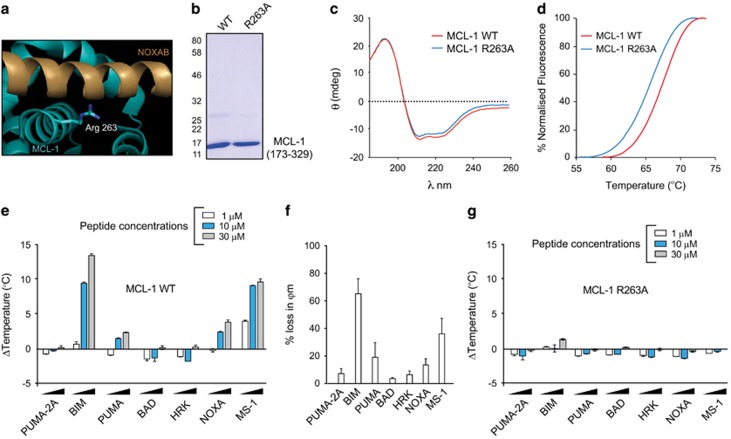
Analysis of purified recombinant WT and R263A MCL-1 (E173-R329) proteins. (**a**) Modelled binding mode of NOXAB peptide engaged with MCL-1 BH3 domain (PDB: 2JM6) with the critical Arg 263 side-chain highlighted. (**b**) SDS-PAGE and Coomassie blue staining of gel filtered, purified recombinant WT and R263A MCL-1 (2 *μ*g) proteins. (**c**) Circular dichroism spectra showing similar secondary structures of WT (red) and R263A (blue) MCL-1 (E173-R329) proteins. (**d**) TSA comparing thermal denaturation (unfolding) profiles for WT (red) and R263A (blue) MCL-1 (E173-R329) proteins. (**e**) Validation of a MCL-1 TSA using synthetic BH3 peptides, demonstrating concentration-dependent binding, characterised by a positive shift of change in the unfolding temperature (ΔTemperature). (**f**) BH3 profiling in H929 cells reveals an enhanced loss in mitochondrial membrane potential (*ϕ*m) following exposure to BIM, PUMA, NOXA and MS-1 peptides, consistent with their ability to bind to MCL-1. (**g**) ΔTemperature (Δ*T*_m_) values for R263A MCL-1 confirms little, or no, detectable binding of MCL-1 to the indicated synthetic peptides. Mean Δ*T*_m_ values±S.D. (*n*=2) were calculated by subtracting the control *T*_m_ value (buffer, no peptide) from the measured *T*_m_ value. The TSA graphs are plotted as duplicate data points (0.3 °C separation per point) and are representative of a single experiment performed in duplicate, which was repeated at least three times

Since *in vitro* binding assays for these drugs were carried out using different assays under distinct experimental conditions, it is impossible to correlate discrepancies between reported *in vitro* binding affinities (Figure 1a) and quantified cellular effects (Figure 1b) without introducing some form of standardisation. To overcome this technical challenge, we developed and validated a new *in vitro* assay that enabled us to compare, under identical experimental conditions, the relative effects of putative MCL-1 inhibitors. DSF is a biophysical method that can be used in a thermostability assay (TSA) workflow to monitor shifts in unfolding parameters as a measure of ligand binding affinity.^17, 18, 19, 20, 21, 22^ We initially isolated a recombinant truncated version of human MCL-1 (amino acids 172-329, corresponding to the BH1-3 domains) as well as a R263A mutant to serve as negative control, as the conserved arginine at position 263 of MCL-1 is critical for ligand and substrate binding in all BCL-2 family members (Figures 2a and b).^23^ Both wild-type (WT) and R263A MCL-1 exhibited similar folded secondary structure profiles (assessed by circular dichroism) and thermal unfolding profiles (assessed by DSF; Figures 2c and d), although R263A MCL-1 (Tm ~66 °C) was slightly less stable than WT MCL-1 (Tm ~67.5 °C). To validate whether recombinant MCL-1 proteins were functionally relevant for screening putative MCL-1 inhibitors, we assessed the ability of different synthetic BH3 peptides to bind WT and R263A MCL-1. As expected, the MCL-1 specific synthetic peptide, MS-1, and a peptide corresponding to the BH3 domain of BIM, were both very efficient MCL-1 binding partners, with temperature shifts of ~10 °C recorded at equimolar peptide and MCL-1 protein concentration. In contrast, PUMA and NOXA peptides showed more modest MCL-1 binding signals, even at high concentrations (Figure 2e). Under identical conditions, no detectable MCL-1 binding was observed for BAD (which binds specifically to BCL-2, BCL-X_L_ and BCL-w but not MCL-1), HRK (BCL-X_L_-specific) and PUMA-2A (a negative control) synthetic peptides (Figure 2e). These results were entirely consistent with the extent of mitochondrial depolarisation induced by these peptides in a cellular BH3 profiling assay using H929 cells (compare Figures 2e and f). Furthermore, none of the BH3 peptides bound/stabilised the R263A mutated MCL-1 protein (Figure 2g), validating the importance of this highly conserved basic residue for peptide binding, and creating a powerful dual recombinant protein screening system for further mechanistic analysis of MCL-1 ligands.

### DSF identifies A-1210477 as a MCL-1 specific inhibitor

**Figure 3:**
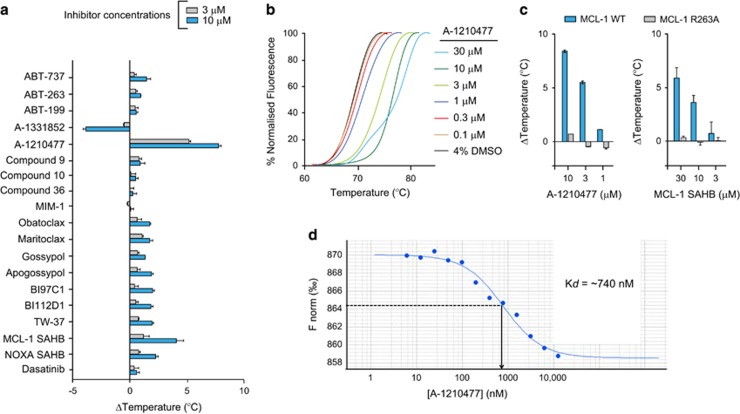
Inhibitor screening using differential scanning fluorimetry (DSF) reveals that A-1210477, but not other BH3 mimetics, exhibit marked MCL-1 binding and thermal stabilisation. (**a**) MCL-1 TSA screen of BH3 mimetics evaluated in the study reveals marked stabilisation of MCL-1 by A-1210477 and MCL-1 SAHB. In contrast, A-1331852 exhibits a negative perturbation (destabilisation) in thermal stability, possibly suggesting either nonspecific binding or an unusual binding mode to MCL-1. (**b**) A-1210477 exhibits a concentration-dependent increase in MCL-1 stabilisation. (**c**) A-1210477 and MCL-1 SAHB exhibit concentration-dependent binding to WT but not R263A MCL-1. (**d**) MCL-1 protein (173–329) was covalently labelled with the fluorescent red dye NT-647 and binding to A-1210477 analysed using microscale thermophoresis. The TSA graphs are plotted as duplicate data points (0.3 °C separation per point) and are representative of a single experiment performed in duplicate, which was repeated at least three times

Next, we next used DSF to screen 18 compounds that have been reported to be MCL-1 inhibitors, concluding that A-1210477 elicited the most marked thermal shift (Δ*T*_m_ value of 6–8 °C), in contrast to the weak thermal shifts observed with other inhibitors of <2 °C, which is within the cut-off for a weak or nonspecific binding event, as defined for ligand binding to protein kinases and bromodomains.^22^ MCL-1 SAHB was the next-best inducer of MCL-1 stabilisation, exhibiting a thermal shift of ~4 °C at a concentration of 10 *μ*M (Figure 3a). Interestingly, the BCL-X_L_ ligand A-1331852 consistently destabilised MCL-1 protein at concentrations of 10 *μ*M and above (Figure 3a). This effect was specific to MCL-1 protein, and did not occur with any other proteins tested. However, destabilisation also occurred with MCL-1 R263A, suggesting a nonspecific interaction elsewhere on the MCL-1 protein (data not shown). In contrast, MCL-1 thermal shifts with A-1210477 and MCL-1 SAHB were both concentration-dependent and completely abolished in MCL-1 R263A (Figure 3b and c). Finally, we directly measured the affinity of the interaction between MCL-1 and A-1210477 using Microscale Thermophoresis (MST), which confirmed a sub-micromolar *K*_d_ value of ~740 nM (Figure 3d). Taken together, these data strongly suggest that A-1210477 is a bona fide MCL-1 inhibitor in a cellular context (Figure 1b) and represents the most potent MCL-1 ligand evaluated using our thermal shift assay, with concentration-dependent Δ*T*_m_ values suggestive of high-affinity (nM) binding, corroborated by MST analysis.^24, 25, 26^

### A-1210477 synergises with a BCL-X_L_ inhibitor to induce the intrinsic apoptotic pathway

**Figure 4:**
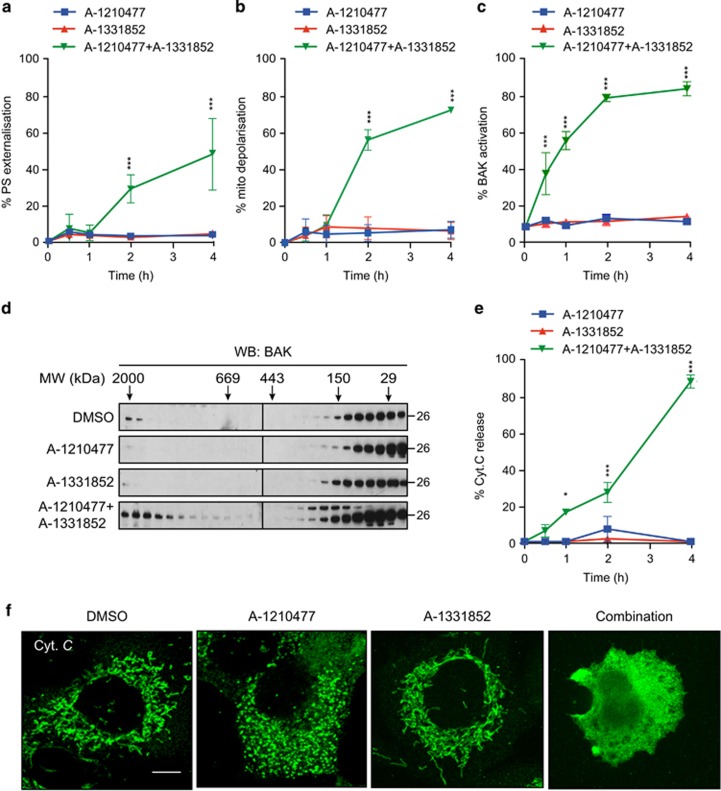
Inhibition of both BCL-X_L_ and MCL-1 is required to release mitochondrial cytochrome *c* in H1299 cells. (**a**) H1299 cells were exposed to A-1210477 (10 *μ*M), A-1331852 (100 nM) or both compounds. Only the combined treatment lead to a time-dependent increase in apoptosis, as assessed by (**a**) PS externalisation, (**b**) mitochondrial depolarisation, (**c**) BAK activation, (**d**) BAK oligomerisation and (**e** and **f**) mitochondrial cytochrome *c* release. For cytochrome *c* release, the cells were immunostained with cytochrome *c* antibody (scale bar, 10 *μ*m) and ~100 cells for each treatment in three independent experiments were quantified for the loss of mitochondrial cytochrome *c*. Statistical analysis was conducted using one-way ANOVA and *P*-values depicted as *P⩽0.05 and ***P⩽0.001

Next, we wished to confirm whether A-1210477 could synergise with distinct BH3 mimetics to induce apoptosis in cells that depend on another BCL-2 family member, in addition to MCL-1. Individual exposure of H1299 cells, which depend on both MCL-1 and BCL-X_L_ for survival,^27, 28^ to either A-1210477 or A-1331852 (a specific BCL-X_L_ inhibitor) had little effect on apoptosis, whereas a combination of the two compounds resulted in a marked induction of apoptosis (Figure 4a). This was accompanied by a concomitant loss of the mitochondrial membrane potential, activation and oligomerisation of BAK and release of mitochondrial cytochrome *c* (Figures 4b–f).

### A-1210477 induces DRP-1-mediated mitochondrial fission

**Figure 5:**
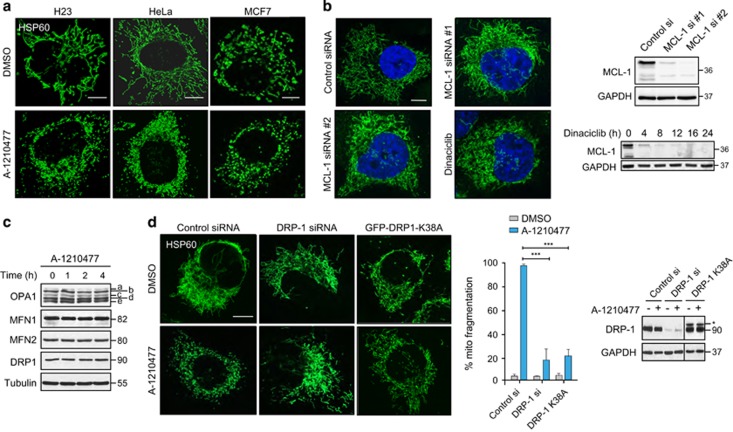
A-1210477 induces extensive mitochondrial fragmentation in a DRP-1-dependent manner. (**a**) H23, HeLa and MCF7 cells exposed to A-1210477 (10 *μ*M) for 2 h resulted in extensive mitochondrial fragmentation, as assessed by immunostaining with HSP60 antibody (scale bar, 10 *μ*m). (**b**) H1299 cells were either transfected with control siRNA, two different MCL-1 siRNAs for 72 h or exposed to Dinacilib (30 nM) for 4 h and assessed for mitochondrial integrity by immunostaining with HSP60 antibody (scale bar, 10 *μ*m). The blots show knockdown efficiency of MCL-1 siRNAs and downregulation of MCL-1 by Dinaciclib. (**c**) Western blot of H1299 cells exposed to A-1210477 (10 *μ*M) for the indicated times revealed no major changes in total expression levels of mitochondrial fission or fusion proteins. Tubulin was used as a loading control. (**d**) H1299 cells were either transfected with control siRNA, DRP-1 siRNA or GFP-DRP-1 K38A plasmid, then exposed to A-1210477 (10 *μ*M) for 2 h and the decrease in the extent of mitochondrial fragmentation was quantified by assessing ~100 cells from each treatment in three independent experiments (scale bar, 10 *μ*m). Statistical analysis was conducted using one-way ANOVA (***P⩽0.001). The blots show the knockdown efficiency of DRP-1 siRNA and the overexpression of GFP-DRP-1 K38A (denoted by*)

The exposure of H1299 cells to A-1210477 resulted in a highly fragmented mitochondrial network, whereas the cells exposed to A-1331852 retained normal mitochondrial structure (Figure 4f). A-1210477-mediated mitochondrial fragmentation was observed in multiple cancer cell lines (Figure 5a) but these mitochondrial changes did not result in apoptosis (data not shown). Interestingly, neither genetic MCL-1 silencing (RNA interference) nor transcriptional MCL-1 repression (using the CDK inhibitor dinaciclib) phenocopied A-1210477-mediated mitochondrial fragmentation (Figure 5b), strongly suggesting that these effects occurred in a MCL-1-independent manner. As the length and continuity of the filamentous mitochondrial network is regulated by specific fission and fusion proteins, we speculated that A-1210477 may alter expression levels of mitochondrial fission and/or fusion GTPases. However, A-1210477-mediated mitochondrial fragmentation was not accompanied by a loss of fusion proteins, such as MFN1 or MFN2 and/or enhanced OPA1 proteolysis (Figure 5c), almost certainly excluding a fusion defect. In contrast, inactivation of the fission protein, DRP-1 using either a siRNA or a dominant-negative DRP-1 mutant (K38A) efficiently reduced mitochondrial fragmentation following A-1210477 exposure (Figure 5d), confirming the absolute requirement of DRP-1 in A-1210477-mediated mitochondrial fission.

### DRP-1-is required for BH3 mimetic-mediated apoptosis

**Figure 6:**
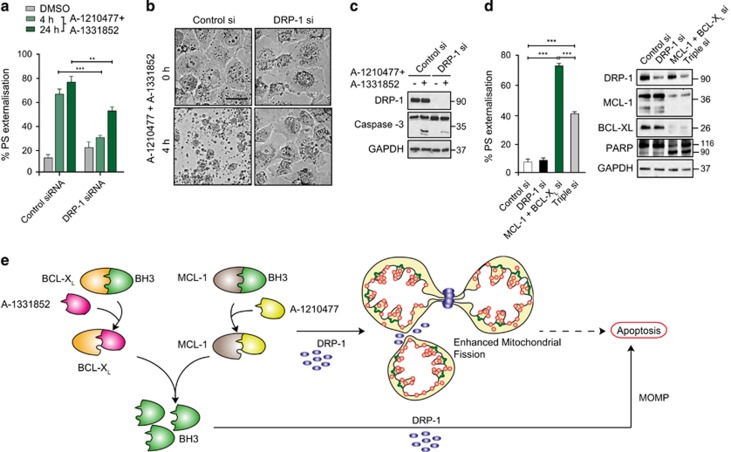
BH3 mimetics induce apoptosis in a DRP-1-dependent manner. (**a**) H1299 cells, transfected with DRP-1 siRNA and exposed to a combination of A-1210477 (10 *μ*M) and A-1331852 (100 nM) demonstrate a marked decrease in apoptosis at 4 and 24 h. Statistical analysis was conducted using one-way ANOVA (**P<0.005, ***P⩽0.001). (**b**) Bright-field microscopy of H1299 cells transfected with control or DRP-1 siRNA and treated with A-1210477 (10 *μ*M) and A-1331852 (100 nM) for 4 h reveals the extent of DRP-1-dependent cell death (scale bar, 30 *μ*m). (**c**) Immunoblots showing the knockdown efficiency of DRP-1 siRNA as well as its anti-apoptotic effects, as evident from the decrease in the processing of pro-caspase-3 following DRP-1 downregulation. (**d**) H1299 cells were transfected with siRNAs against both BCL-X_L_ and MCL-1 in the absence or presence (Triple si) of DRP-1 siRNA for 36 h and the extent of apoptosis assessed by PS externalisation. Blots showing the knockdown efficiency of the siRNAs and the anti-apoptotic effects associated with DRP-1 deficiency, as evident from the decrease in the processing of the caspase substrate, PARP. Statistical analysis was conducted using one-way ANOVA (***P⩽0.001). (**e**) Scheme representing the distinct functions of A-1210477 in apoptosis induction (by releasing BH3-only members from the hydrophobic groove and causing mitochondrial outer membrane permeabilisation; MOMP) as well as increased mitochondrial fission in a DRP-1 dependent manner

Although A-1210477-mediated mitochondrial fragmentation did not directly result in apoptosis, it remained possible that such major structural alterations could sensitise cells to apoptosis. Interestingly, downregulation of DRP-1 not only prevented mitochondrial fragmentation but also markedly reduced the extent of apoptosis, following exposure to the combination of A-1210477 and A-1331852 (Figures 6a–c). Silencing the dual expression of BCL-X_L_ and MCL-1 resulted in extensive apoptosis (Figure 6d), independent of any mitochondrial fragmentation (data not shown). DRP-1 deficiency also diminished apoptosis in these cells (Figure 6d) suggesting that the protective effect of DRP-1 siRNA was independent of mitochondrial fragmentation, and occurred most likely due to its known effects on mitochondrial outer membrane permeabilisation (MOMP; Figure 6e).^29^ Taken together, these results strongly suggested that DRP-1 was required both for BH3 mimetic-induced mitochondrial fragmentation as well as apoptosis.

## Discussion

Several promising small molecule inhibitors reported to target MCL-1 *in vitro* have been largely ineffective in a cellular context.^27^ The failure to convert a ‘high-affinity' (often reported as nM) *in vitro* MCL-1 binding affinity into an enhanced apoptosis phenotype in a physiological context might be partly due to the lack of accessibility of the drugs at sites of action, thus rendering it more difficult to treat solid tumours than circulating B cells in lymphoid malignancies. Alternatively, high expression levels of additional BCL-2 family members in solid tumours could result in functional target redundancy, raising the prospects of a requirement for pan-BCL-2 family inhibition as a therapeutic strategy. With the exception of A-1210477, most of the other putative MCL-1 inhibitors tested here induced cell death in a MCL-1-dependent cell line only after prolonged cellular exposure (Figure 1b). This is in stark contrast to the rapid and extensive apoptosis observed with other potent clinical BH3 mimetics, such as ABT-263 and ABT-199.^2, 27, 30^ Indeed, the induction of cell death at these later times might be attributed to either other indirect measures of downregulating MCL-1 (such as transcriptional or translational repression and NOXA upregulation) or other off-target effects associated with these inhibitors.^27, 31^

In this study, we developed a novel DSF approach to evaluate the effects of a panel of putative MCL-1 inhibitory ligands, characterising A-1210477 as the only compound in our panel that bound MCL-1 in a R263-dependent manner and induced concentration-dependent apoptosis (Figures 1, 2, 3). Although MCL-1 SAHB also bound MCL-1 rather specifically (Figure 3), it was inefficient in inducing apoptosis in a cellular context, either due to weak target engagement or weak cell permeability. In contrast to most other putative inhibitors tested, A-1210477 also readily perturbed the thermal-induced unfolding of MCL-1 at concentrations consistent with high-affinity binding to MCL-1 (Figure 3), providing strong support for the exploitation of this assay for the identification and benchmarking of other MCL-1 ligands. We believe that this simple and rapid method could readily be exploited to compare and contrast both potency and mechanistic aspects of compound binding for MCL-1, circumventing issues associated with data comparison and poor reliability demonstrated for more complex fluorescence assays (most of which involve FRET or FP).

Indeed, there is now an urgent need for specific and potent small molecule inhibitors of MCL-1 that efficiently target cancer cells while sparing physiological cellular functions of MCL-1. While this manuscript was in preparation, a new single-digit nanomolar MCL-1 inhibitor, S63845, which induces rapid apoptosis in MCL-1-dependent cells, was reported.^32^ It will be interesting to assess whether S63845 exhibits mitochondrial structural changes similar to those observed with A-1210477. Nonetheless, of all the compounds tested in the current study, only A-1210477 was prominent in terms of MCL-1 binding in DSF assay and rapid induction of apoptosis in cellular assays. However, A-1210477 still requires micromolar concentrations in a cellular context to exert apoptotic effects, and is predicted to lack the potency for *in vivo* efficacy, where nanomolar compounds are likely to be a prerequisite. The discovery of high-affinity compounds such as S63845 that reportedly possess this higher cellular efficacy in tumour models might help alleviate this issue in the clinic.

## Materials and Methods

### Cell culture

The H929 cells from ECACC (Salisbury, UK), H23 and H1299 cells from ATCC (Middlesex, UK) were cultured in RPMI 1640 medium supplemented with 10% fetal calf serum (FCS) from Life Technologies, Inc., (Paisley, UK). HeLa and MCF7 from ATCC were cultured in DMEM medium supplemented with 10% FCS (all from Life Technologies, Inc.).

### BCL-2 family inhibitors

ABT-737, ABT-263, ABT-199, A-1331852 and A-1210477 were kindly provided by AbbVie Inc. (North Chicago, IL, USA). Compounds 10, 20, 21, 22, 36, 37 and 41 were provided by Dr. Z Nikolovska-Coleska (University of Michigan, Ann Arbor, MI, USA).^8^ Compound 9 was custom-synthesised and purchased from Molport (Riga, Latvia).^6^ MIM-1 and the stapled peptides against MCL-1 and NOXA were kindly provided by Dr. L Walensky (Dana-Farber Cancer Institute, Boston, MA, USA).^7, 16^ Maritoclax was provided by Professor H-G Wang (Pennsylvania State University College of Medicine, Hershey, PA, USA).^10^ Apogossypol, Sabutoclax and BI112D1 were provided by Professor M. Pellecchia (Sanford-Burnham Institute, La Jolla, CA, USA).^11, 12^ Gossypol, Obatoclax and TW-37 were obtained from Selleck Chemicals Co. (Houston, TX, USA).

### Reagents and plasmids

Peptides for BIM (MRPEIWIAQELRRIGDEFNA), BID (EDIIRNIARHLAQVGDSMDRY), NOXA (AELPPEFAAQ LRKIGDKVYC), PUMA (EQWAREIGAQLRRMADDLNA), HRK (WSSAAQLTAARLKALGDE LHQ), MS-1 (RPEIWMTQGLRRLGDEINAYYAR), BAD (LWAAQRYGRELRRMSDEFEGSFKGL) and PUMA-2 A (EQWAREIGAQARRMAADLNA) were from New England Peptide (Gardner, MA, USA) or GenScript (Piscataway, NJ, USA). Antibodies against MCL-1, BAK and GAPDH from Santa Cruz Biotechnology (Santa Cruz, CA, USA); OPA1, DRP-1, HSP60 and Cytochrome *C* from BD Biosciences (Oxford, UK); Tubulin, MFN1, MFN2, Caspase-3, MCL-1 (RC-13) from Abcam (Cambridge, UK); BAK (Ab-1) from Merck Chemicals Ltd (Nottingham, UK) were used. For bacterial expression of recombinant proteins, a human MCL-1 dsDNA corresponding to amino acids E173- R329 was amplified using the primer sets, 5′-AAGTTCTGTTTCAGGGCCCGGAGTTGTACCGGCAGTCG-3′ and 5′-ATGGTCTAGAAAGCTTTACCTGATGCCACCTTCTAGGTC-3′ and cloned into pOPIN F (OPPF-UK, Oxford, UK) to generate N-terminally His6-tag fusion protein. This was used as a template to generate the R263A mutant using site-directed mutagenesis with the primer sets, 5′-CGTAACAAACTGGGGCGCGATTGTGACTCTC-3′ and 5′-GAGAGTCACAATCGCGCCCCAGTTTGTTACG-3′. GFP-DRP-1 K38A plasmid was described previously.^33^ All other reagents, unless mentioned otherwise, were from Sigma-Aldrich Co. (St. Louis, MO, USA).

### Protein purification

WT MCL-1 fusion protein (N-terminal His6-tag fused to amino acids Glu173-Arg329, containing the BH3, BH1 and BH2 domains) and an R263A substitution in the WGRIV motif found in the MCL-1 BH1 domain were produced in BL21 (DE3) pLysS *E. coli* cells (Novagen, Nottingham, UK), induced with 0.4 mM IPTG for 18 h at 18 °C and purified by immobilised metal affinity chromatography (IMAC) using Ni-NTA agarose (Qiagen, Manchester, UK) and size exclusion chromatography using a HiLoad 16/600 Superdex 200 column (GE Healthcare, Chicago, IL, USA), equilibrated in 50 mM Tris HCl, pH 7.4, 100 mM NaCl, 10 % (v/v) glycerol and 1 mM DTT. Secondary structure compositions of WT and R263A MCL-1 proteins (0.9 mg/ml) were analysed by circular dichroism in the far UV range (180–260 nm) using a Jasco 1100 CD spectrometer with a path length of 0.1 cm, following buffer exchange into 10 mM sodium phosphate (pH 7.4) and 25 mM NaF.

### DSF assays

Thermal shift assays (TSA) were performed using a StepOnePlus Real-Time PCR machine (Life Technologies, Paisley, UK) using Sypro-Orange dye (Invitrogen, Paisley, UK). Thermal ramping (0.3 °C per min between 25 and 94 °C) was used to generate thermal denaturation curves for purified MCL-1 proteins (10 *μ*M) in the presence or absence of the indicated concentrations of ligand (final DMSO concentration 4% v/v). Data were processed using the Boltzmann equation to generate sigmoidal denaturation curves, and average *T*_m_/Δ*T*_m_ values calculated as previously described,^26^ using GraphPad Prism software.

### Microscale thermophoresis

MCL-1 protein (173–329) was covalently labelled with the fluorescent red dye NT-647, which reacts with primary amines present on MCL-1 Lys residues to form a stable dye–protein conjugate before analysis. The concentration of MCL-1 was kept constant in the assay and the concentration of the unlabelled A-1210477 ligand diluted in buffer was varied between 6 nM and 200 *μ*M. The assay was performed after loading into capilliaries 25 mM HEPES, pH 7.4 containing 0.05% Tween-20. MST analysis was performed using the Monolith NT.115 instrument and data were plotted using Monolith software.

### BH3 profiling and flow cytometry

BH3 profiling was carried out as previously described.^34^ In brief, the cells were permeabilised with digitonin (0.002 %) in DTEB buffer (10 mM HEPES, 135 mM Trehalose, 20 *μ*M EDTA, 20 *μ*M EGTA, 5 mM succinic acid, 0.1 % BSA, 50 mM potassium chloride, pH 7.5) containing oligomycin (10 *μ*g/ml) and incubated for 2 h with varying concentrations of BH3 peptides. The loss of mitochondrial membrane potential (*ϕ*m) was measured by observing the loss of TMRE (200 nM) using an Attune NxT flow cytometer (ThermoFisher Scientific, Paisley, UK). The extent of apoptosis in cells following different treatments was quantified by FACS following staining of the cells with Annexin V-FITC and propidium iodide to measure phosphatidylserine externalisation, as previously described.^33^ For monitoring BAK activation, the cells pre-treated with Z-VAD(OMe).fmk (Selleck Chemicals) for 30 min were exposed to the indicated drugs, fixed in 1 % (w/v) paraformaldehyde and stained with conformation-specific AP-1 BAK antibody (1 *μ*g/ml), corresponding fluorophore- conjugated secondary antibody and quantified by FACS.

### siRNA knockdowns

For transient transfections, the cells were transfected using TransIT-LT-1 transfection reagent (Mirus Bio LLC, Madison, WI, USA) and left for 48 h, according to the manufacturer's instructions. For siRNA knockdowns, the cells were transfected for 72 h with DRP-1 siRNA (S104274235; Qiagen), MCL-1 siRNA (s8585; Ambion and SI02781205; Qiagen) or BCL-XL siRNA (s1920; Ambion) using Interferin Reagent (Polyplus transfection Inc, NY, USA), according to the manufacturer's protocol.

### Gel filtration and western blotting

For gel filtration experiments, the cells were lysed in CHAPS lysis buffer containing 1 % CHAPS, 20 mM Tris HCl (pH 8), 150 mM NaCl, 20 *μ*M MG132 and protease inhibitor cocktail, incubated on ice for 30 mins, and centrifuged at 14 000 × *g* for 3 min at 4 °C. The pellet was discarded and supernatant (500 *μ*l; 25 mg of protein per ml) was injected onto both Superose 6 and Superdex S200 size exclusion columns (GE Healthcare) and eluted using a running buffer containing 50 mM Tris HCl (pH 8), 150 mM KCl, 1 mM EDTA, 5 % glycerol, 1 mM DTT and 1 mM PMSF. 500 *μ*l fractions eluted from the columns were collected for immunoblotting. Western blotting was carried out according to standard protocols. Briefly, 50 *μ*g of total protein lysate was subjected to SDS-PAGE electrophoresis. Subsequently proteins were transferred to nitrocellulose membrane and protein bands visualised with ECL reagents (GE Healthcare).

### Microscopy

Bright-field microscopy of cells was performed using EVOS FLOID cell imaging station (ThermoFisher Scientific). For immunofluorescent staining, the cells grown on coverslips were fixed with 4 % (w/v) paraformaldehyde, permeabilised with 0.5 % (v/v) Triton X-100 in PBS and followed by incubations with primary antibodies, the appropriate fluorophore-conjugated secondary antibodies, mounted on glass slides and imaged using a 3i Marianas spinning disk confocal microscope, fitted with a Plan-Apochromat × 63/1.4 NA Oil Objective, M27 and a Hamamatsu ORCA-Flash4.0 v2 sCMOS Camera (all from Intelligent Imaging Innovations, GmbH, Gottingen, Germany).

### Statistical analysis

For time-course studies, a two-way ANOVA was performed and other studies were analysed for statistical significance with one-way ANOVA and the asterisks depicted correspond to the following *P*-values: *P⩽0.05, **P⩽0.005 and ***P⩽0.001.
